# Diagnosis and management of gastrointestinal complications in adult cancer patients: 2017 updated evidence-based guidelines of the Infectious Diseases Working Party (AGIHO) of the German Society of Hematology and Medical Oncology (DGHO)

**DOI:** 10.1007/s00277-017-3183-7

**Published:** 2017-11-24

**Authors:** M. Schmidt-Hieber, J. Bierwirth, D. Buchheidt, O. A. Cornely, M. Hentrich, G. Maschmeyer, E. Schalk, J. J. Vehreschild, Maria J. G. T. Vehreschild, for the AGIHO Working Group

**Affiliations:** 1 Clinic for Hematology, Oncology, Tumor Immunology and Palliative Care, HELIOS Klinikum Berlin-Buch, Berlin, Germany; 2 Deutsches Beratungszentrum für Hygiene, BZH GmbH, Freiburg, Germany; 3 3rd Department of Internal Medicine - Hematology and Oncology - Mannheim University Hospital, University of Heidelberg, Heidelberg, Germany; 4 1st Department of Internal Medicine, University of Cologne, Cologne, Germany; 5 German Center for Infection Research (DZIF), partner site Bonn-Cologne, Cologne, Germany; 6 Clinical Trials Centre Cologne, ZKS Köln, University of Cologne, Cologne, Germany; 7 Department III for Internal Medicine, Hematology and Oncology, Rotkreuzklinikum München, Munich, Germany; 8 Department of Hematology, Oncology and Palliative Care, Ernst-von-Bergmann Klinikum, Potsdam, Germany; 9 Department of Hematology and Oncology, Medical Center, Otto-von-Guericke University, Magdeburg, Germany; 10 1st Department of Internal Medicine, Hospital of the University of Cologne, Kerpener Str. 62, 50937 Köln, Germany

**Keywords:** Abdominal complications, Colitis, Diarrhea, Chemotherapy, Cancer, Infection

## Abstract

Cancer patients frequently suffer from gastrointestinal complications. In this manuscript, we update our 2013 guideline on the diagnosis and management of gastrointestinal complications in adult cancer patients by the Infectious Diseases Working Party (AGIHO) of the German Society of Hematology and Medical Oncology (DGHO). An expert group was put together by the AGIHO to update the existing guideline. For each sub-topic, a literature search was performed in PubMed, Medline, and Cochrane databases, and strengths of recommendation and the quality of the published evidence for major therapeutic strategies were categorized using the 2015 European Society for Clinical Microbiology and Infectious Diseases (ESCMID) criteria. Final recommendations were approved by the AGIHO plenary conference. Recommendations were made with respect to non-infectious and infectious gastrointestinal complications. Strengths of recommendation and levels of evidence are presented. A multidisciplinary approach to the diagnosis and management of gastrointestinal complications in cancer patients is mandatory. Evidence-based recommendations are provided in this updated guideline.

## Introduction

Abdominal complications are a frequent problem in patients with hematological malignancies or solid tumors. In 2013, we published the first version of “Diagnosis and management of gastrointestinal complications in adult cancer patients: evidence-based guidelines of the Infectious Diseases Working Party (AGIHO) of the German Society of Hematology and Medical Oncology (DGHO)”, one of the first comprehensive, practical, and evidence-based guidelines covering the epidemiology, pathophysiology, diagnosis and treatment of most non-infectious and infectious complications as well as the corresponding hygiene measures [1]. The present update takes the evolving basis of evidence into consideration. In addition, the section on infectious complications caused by parasites has been considerably expanded in response to the increasing mobility of our patients. Whenever possible, pre-existent recommendations from other guideline panels were incorporated into this overview.

## Methods

Sub-topics of this guideline were assigned to members of the AGIHO and a literature search was performed in PubMed, Medline, and Cochrane databases. The strength of recommendation and the quality of evidence for major therapeutic strategies were categorized using the current criteria of the European Society for Clinical Microbiology and Infectious Diseases (ESCMID, Table 1) which include an index to the level II recommendations, where appropriate [2].

**Table 1:** Categories of evidence—ESCMID criteria [2]

Category, Grade	Definition
Strength of recommendation	
A	AGIHO *strongly* supports a recommendation for use
B	AGIHO *moderately* supports a recommendation for use
C	AGIHO *marginally* supports a recommendation for use
D	AGIHO supports a recommendation *against* use
Quality of evidence	
I	Evidence from at least one properly designed randomized, controlled trial
II	Evidence from at least one well-designed clinical trial, without randomization; from cohort or case-control analytic studies (preferably from more than one center); from multiple time series; or from dramatic results of uncontrolled experiments
III	Evidence from opinions of respected authorities, based on clinical experience, descriptive case studies, or reports of expert committees
Index (for level II quality of evidence, only)	
r	Meta-analysis or systematic review of randomized controlled trials
t	Transferred evidence, i.e., results from different patient cohorts, or similar immune-status situation
h	Comparator group is a historical control
u	Uncontrolled trial
a	Abstract published at an international meeting

Consensus discussions were held on each of the topics. After ratification of all topics by this expert group, recommendations were discussed and ratified by the AGIHO plenary.

Treatment-associated anorexia, nausea, and emesis are not included, as this would exceed the scope of this guideline and has already been addressed elsewhere [3].

## Guideline

### Diarrhea and colitis

**Fig. 1:**
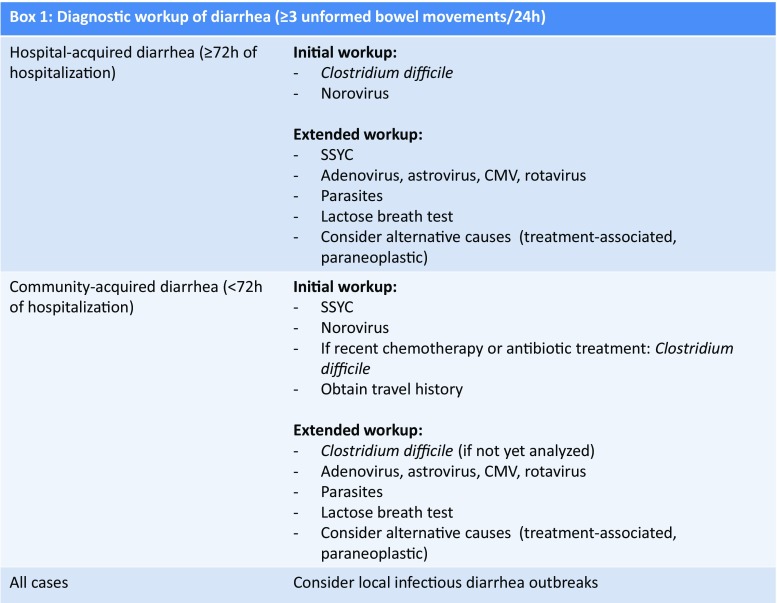
Diagnostic work-up of diarrhea (≥ 3 unformed bowel movements/24 h)

Independent of its cause, diarrhea should always be treated with adequate oral or intravenous fluid and electrolyte replacement **(AIII)**. Patients should be observed for signs of malnutrition and/or catabolic state. If indicated, enteral or parenteral electrolytes, carbohydrates, lipids, amino acids, protein, and vitamins should be supplemented (**AIII**). Figure 1 provides important facts on the diagnostic work-up of diarrhea in cancer patients. Commonly, repeat testing for the same pathogen should not be performed to avoid false positive results [4, 5].

#### Non-infection-related diarrhea and colitis

##### Paraneoplastic diarrhea

Paraneoplastic diarrhea is a rare phenomenon which may be triggered by a variety of pathophysiological mechanisms. Secretion of vasoactive intestinal polypeptide (VIP), as typically observed in patients with non-β islet cell tumors of the pancreas, may cause watery diarrhea, hypokalemia, and hypochlorhydria [6]. Flush and diarrhea are the typical symptoms of serotonin-producing carcinoid tumors [7]. Other hormones that may cause paraneoplastic diarrhea include glucagon (glucagonoma), gastrin (gastrinoma or hepatocellular carcinoma), somatostatin (somatostatinoma or pheochromocytoma), and the prostaglandins (hepatocellular carcinoma) [8–12]. In association with small cell lung carcinoma, antibodies directed against neuronal proteins may cause autonomic neuropathy and diarrhea [13]. In patients with thymoma, diarrhea as part of a graft-versus-host-disease-like reaction has been described [14, 15].

In most cases of paraneoplastic diarrhea, diagnosis and treatment of the underlying disease is considered the only effective measure to reduce diarrhea. If a well-differentiated neuroendocrine neoplasia (NEN) has been diagnosed and patients present with typical symptoms of a functional NEN such as diarrhea and flush, treatment with somatostatin analogs is indicated as discussed elsewhere [16].

##### Therapy-associated diarrhea

In cancer patients, factors related to toxic effects of chemotherapy are the most common cause of abdominal complications*.* 5-fluorouracil, irinotecan, capecitabine, anthracyclines, and a number of small molecules and monoclonal antibodies have been associated with therapy-associated diarrhea [17–25]. Previous studies have reported incidence rates of diarrhea in 27–76% of neutropenic patients. In only 5–17% of these cases, an infectious agent was identified as the cause of diarrhea, suggesting primarily toxicity-related symptoms [17, 26–28]. Disruption of the gastrointestinal microflora after administration of antibiotics may result in osmotic diarrhea due to alterations in carbohydrate metabolism and impaired absorption of short-chain fatty acids in 5–62% of patients [29–31]. In 7–50% of these cases, overgrowth with *Clostridium difficile* may ensue, leading to *C. difficile*-associated diarrhea [32, 33].

Chemotherapy-associated lactose intolerance presenting as diarrhea, bloating, and malabsorption has also been discussed as a cause of non-infectious diarrhea in cancer patients. While up to 35% of patients present with an abnormal lactose breath hydrogen test during chemotherapy, only up to 11% became symptomatic. Generally, test results returned to normal after completion of chemotherapy [34, 35].

Radiotherapy involving the gastrointestinal tract may cause severe mucosal bowel damage resulting in acute or chronic diarrhea. Symptoms usually peak about 7–14 days after initiation of irradiation and may be intensified by combination treatment with chemotherapy. In some patients, surgical resection may result in impairment of physiological gastrointestinal function with diarrhea developing because of accelerated gastric and intestinal transit times, bacterial overgrowth, and altered secretion and absorption of bile acids.

After exclusion of an infectious cause of diarrhea (see Fig. 1), loperamide is recommended for first-line treatment of non-infectious diarrhea (initial dose 4 mg, followed by 2 mg po after each unformed bowel movement, maximum daily dose is 16 mg) [36–39] (**AII**_**u**_). Patients with severe diarrhea persisting for more than 48 h despite administration of antimotility agents should be hospitalized [36] (**AIII**). Of note, in long-term neutropenic patients, overdosage of antimotility agents may lead to iatrogenic ileus with an increased risk of bacteremia [27].

In patients failing to respond to loperamide, octreotide may be considered with a starting dose of 100 μg tid sc [40–42] (**BII**_**u**_). In patients not responding to the initial dosage, dose increases until symptom control are recommended [40–44] (**AIII**). An alternative might be the administration of psyllium seeds, although this approach has not been evaluated in patients with chemotherapy-associated diarrhea [45, 46] (**BII**_**t**_). Further options include diphenoxylate plus atropine and opiates such as paregoric tincture of opium, codeine, and morphine [36] (**BIII**). Primary prevention using octreotide long acting release (LAR) has been evaluated in two randomized controlled trials, but did not prove efficacious [47, 48] (**DI**). The efficacy of glutamine in preventing treatment-associated diarrhea was assessed in a meta-analysis comprising eight heterogeneous small to medium-sized randomized controlled trials. According to this analysis, glutamine significantly reduced duration of diarrhea, but not severity [49]. Another randomized controlled trial showing no advantage for glutamine was not included into this meta-analysis [50]. Overall, the available evidence does not support the use of glutamine in this indication (**DI**). Similarly, ReCharge ice cream based on iron-saturated lactoferrin and anhydrous milk failed to control treatment-associated diarrhea in a randomized controlled trial [51] (**DI**).

Concerning diarrhea associated with specific substances, budesonide or neomycin prophylaxis for late-onset diarrhea after treatment with irinotecan showed no significant advantage [52, 53] (**DI**). However, addition of budesonide [24] (**BII**_**u**_) or acetorphan [25] (**BII**_**u**_) to loperamide in the treatment of manifest irinotecan-associated diarrhea was effective in two small clinical trials. In contrast, preventive calcium aluminosilicate clay did not reduce incidence and severity of diarrhea [54] (**DI**).

5-FU-associated diarrhea was targeted by a small randomized pilot study in which lafutidine, a second generation histamine H2 receptor antagonist, or placebo was administered to ten patients with gastric adenocarcinoma. While the results were promising with respect to reduction of diarrhea and nausea, the limited sample size does not allow for generalization [55] (**CI**). Due to the lack of evaluable data, an expert panel discussed and published treatments for idelalisib-associated diarrhea and recommended budesonide or other oral or intravenous steroids [56] (**BIII**).

Concerning chemotherapy-associated lactose intolerance, we do not recommend dietary restriction of milk products, unless clinical symptoms of lactose intolerance are observed after ingestion of milk products [34, 35] (**BII**_**u**_).

A large number of trials assessing the protective effect of prophylactic probiotic treatment to avoid antibiotic-associated diarrhea have been conducted. Studies in immunocompetent patients suggest a protective effect for *Saccharomyces boulardii*, *Lactobacillus rhamnosus*, and a combination of *L. casei*, *L. bulgaricus*, and *S. thermophilus* [32, 57, 58]. A recent review identified 11 studies in patients with cancer and concluded that the severity and frequency of diarrhea may be reduced by use of probiotics. However, it was also underlined that more studies were needed to assess effect size and safety aspects, as there were five cases of probiotic-related bacteremia or fungemia [59] (**CII**_**t,r**_). Recommendations on therapy-associated diarrhea are summarized in Table 2.

**Table 2:** Treatment-related diarrhea

Clinical situation	Intention	Intervention	SoR	QoE	Reference	Comments
Treatment-associated diarrhea	Primary prevention	Octeotride LAR	D	I	[47, 48]	No sufficient evidence to recommend the use of octreotide LAR for secondary prevention
		Glutamine	D	I	[49, 50]	
Late-onset diarrhea after irinotecan therapy	Cure	Treatment with loperamide po *plus*				Stop treatment, if no response after 72 h
		Budesonide 3 mg tid po until resolution of symptoms *or*	B	II_u_	[24]	
		Acetorphan 100 mg tid po for 48 h	B	II_u_	[25]	
Late-onset diarrhea after irinotecan therapy	Primary prevention	Prophylaxis with budesonide 3 mg tid po *or*	D	I	[52]	
		Neomycin 500 mg bid po	D	I	[53]	
Treatment-associated diarrhea, 1st line	Cure	Loperamide, initial dose 4 mg, followed by 2 mg po after each unformed bowel movement	A	II_u_	[36–39]	-Only in persisting and severe cases of diarrhea and after exclusion of infectious diarrhea -Careful risk-benefit assessment in neutropenic patients -No further benefit after 16 mg qd -Administer 30 min. before eating for maximum efficacy
Treatment-associated diarrhea, 2nd line	Cure	Octreotide 100 μg tid sc; increase to 500 μg qd if no improvement after 24 h	B	II_u_	[40–44]	-Only in cases of persisting and severe diarrhea and after exclusion of infectious diarrhea -Titration to higher dosages may be considered if no response to 500 μg qd sc -Careful risk-benefit assessment in neutropenic patients -iv administration at 25–50 μg/h possible
	Cure	Psyllium seeds	B	II_t_	[45, 46]	-Only in persisting and severe cases of diarrhea and after exclusion of infectious diarrhea
		Alternatives: Diphenoxylate plus atropine, paregoric tincture of opium, codeine or morphine	B	III		-Only in cases of persisting and severe diarrhea and after exclusion of infectious diarrhea -Careful risk-benefit assessment in neutropenic patients
Chemotherapy-associated lactose intolerance	Prevention	Dietary restriction of milk products	B	II_u_	[34, 35]	Only if clinical signs and symptoms are present
Antibiotic-associated diarrhea	Prevention	Probiotics	C	II_t,r_	[59]	No sufficient safety data in immunocompromised patients available

*SoR* strength of recommendation, *QoE* quality of evidence

#### Infection-related diarrhea and colitis

The diagnosis of infection-related diarrhea should trigger adequate hygiene measures [60] (**AII**). The regular practice of appropriate hand hygiene is considered a cornerstone in the prevention of hospital-acquired infections [61, 62] and has been discussed in detail elsewhere [63]. Table 3 shows recommended hygiene procedures for most common infectious causes of gastroenteritis. Of note, hygiene measures can be subject to local or national legislation which may differ from these recommendations.

**Table 3:** Isolation procedures for the most common causes of infectious diarrhea

Pathogen	SR	GG	M	Infectious material	Stop	SoR	QoE	Comment
*Clostridium difficile*	●	○		Feces	Normalization of clinical symptoms (diarrhea or colitis)	B	III	-Use warm water and plain soap for hand hygiene after patient contact -No precautions for asymptomatically colonized patients -Do not re-test for *C. difficile* toxin to evaluate further necessity of isolation -Gloves and gown only if contact with infectious material or contaminated surfaces
*Salmonella*, *Shigella*, *Yersinia*, *Campylobacter* spp.	●	○		Feces, vomitus, possibly urine	Three negative stool samples	B	III	Gloves and gown only if contact with infectious material or contaminated surfaces
Norovirus	●	●	●	Feces, vomitus	Three negative stool samples	B	III	

● always required, ○ only required under certain circumstances specified in the comment box, *SR* single room, *GG* gloves and gown, *M* mask, *SoR* strength of recommendation, *QoE* quality of evidence

##### Neutropenic enterocolitis

Neutropenic enterocolitis (NEC) is a common chemotherapy-associated complication, particularly in patients with acute leukemia [17, 64–67]. A pooled incidence rate of 5.3% was calculated for hospitalized patients with hematological malignancies, aplastic anemia, or those receiving high-dose chemotherapy for solid tumors [67]. NEC has been associated with mortality rates between < 20 and 82% [65, 68–70]. Administration of high-dose cytarabine plus anthracyclines has been identified as major risk factor. However, many other cytostatic agents and radiotherapy have been identified as triggers of NEC [28, 71–80]. Mucosal barrier damage facilitates infiltration and penetration of the bowel wall by bacteria, viruses, and fungi. From blood cultures drawn during episodes of NEC, Gram-negative *Enterobacteriaceae* were the most frequently documented organisms [17, 27, 66]. A systematic review on fungal infections related to NEC found a pooled frequency of 6.2% [68].

Clinical signs and symptoms include abdominal pain, diarrhea, nausea, and vomiting. In more severe cases rebound tenderness, decreased bowel sounds or muscular guarding may develop. Proposed diagnostic criteria according to Gorschlüter et al. are the presence of fever, abdominal pain, and a bowel wall thickening of more than 4 mm (transversal scan) over more than 30 mm (longitudinal scan) in any segment by ultrasonography or computed tomography (CT) [67].

Since this definition of NEC describes patients at a late pathophysiological stage of intestinal impairment, a clinical definition identifying neutropenic patients at risk of further clinical deterioration due to abdominal complications was recently developed. Neutropenic patients with chemotherapy-associated bowel syndrome (*T* ≥ 37.8 °C and abdominal pain and/or lack of bowel movement for ≥ 72 h) were more likely to suffer complications and death [27].

Non-invasive imaging is generally recommended to confirm the diagnosis of NEC and to exclude bowel wall perforation. Blood cultures, stool cultures, and a *C. difficile* toxin test for exclusion of NEC-associated bacteremia and colitis due to *C. difficile*, respectively, are recommended. Endoscopy to obtain biopsies is discouraged, due to the increased risk of bowel wall perforation.

Conservative therapy is preferred in most cases, consisting of a bland diet, hydration, and an effective pain treatment (**BIII**). In accordance with IDSA guidelines for patients with complicated abdominal infections in non-neutropenic patients [81] and the guideline for antimicrobial therapy of unexplained fever in neutropenic patients of the AGIHO [82], we recommend administration of piperacillin/tazobactam or imipenem/cilastatin or meropenem (**BIII**). There are no studies assessing the effect of additional metronidazole or vancomycin on patient outcome (**CIII**). Empirical antifungal therapy may be discussed if it has not yet been administered for the indication of persistent febrile neutropenia [68, 83, 84] (**BIII**). The use of hematopoietic growth factors might be considered, even though corresponding evidence is not available (**BIII**). Antimicrobial therapy should be administered until resolution of clinical signs and neutropenia. While a surgical consultation should be obtained at an early stage of disease evolution, surgical interventions in the neutropenic and/or thrombocytopenic patient are reserved to severe cases, e.g., patients with bowel wall perforation (**BIII**).

##### *Clostridium difficile* infection

*C. difficile* is the most common cause of healthcare-associated infectious diarrhea and colitis, in both Europe and the USA [85, 86]. In adult patients with cancer, infections due to *C. difficile* (CDI) occur in 5–9% of chemotherapy courses and 5–20% of patients, respectively [27, 28, 66, 87–91].

Binding of *C. difficile* toxins A and B to epithelial cells and subsequent internalization leads to diarrhea by induction of apoptosis [92]. An increase in the frequency of CDI has been reported and attributed to the emergence of a new and hypervirulent strain of *C. difficile*, named NAP1 (synonymous terms are BI, ribotype 027, and toxinotype III) [93–95]. In NAP1 strains, single-base deletion mutations at position 117 of the *tcdC* gene, a downregulator of toxin transcription, lead to disinhibition of toxin A and B production, thus contributing to increased intracolonic toxin levels [96]. The most important risk factors for CDI are antibiotic exposure, advanced age, immunosuppression, and chronic kidney disease. However, other factors such as prolonged length of hospital stay, previous CDI, and use of proton pump inhibitors have also been discussed [97–102].

The most recent ESCMID update on treatment guidance differentiates between non-severe and severe disease, whereas severe disease is indicated by signs of colitis identified by clinical examination or imaging. Alternatively, laboratory markers, i.e., marked leucocytosis (leucocyte count > 15 × 10^9^/L) and/or marked left shift (band neutrophils > 20% of leucocytes), rise in serum creatinine (> 50% above the baseline), elevated serum lactate (≥ 5 mM), or markedly reduced serum albumin (< 30 g/L), may also indicate severe disease. In addition, the guideline suggests classification of patients at an increased risk of developing severe CDI into this same category. Relevant risk factors in this context are age ≥ 65 years, serious comorbidities, intensive care unit admission, and immunodeficiency. Based on the recommendations, cancer patients are unlikely to be classified as non-severe cases, unless they are currently not considered immunocompromised [103].

Clinical signs and symptoms of CDI are diarrhea, fever, abdominal pain, and distension. Presentation ranges from mild diarrhea to fulminant pseudomembranous colitis with paralytic ileus, toxic megacolon, or perforation [88, 90, 104]. Onset of diarrhea may occur at any time during and up to 2 weeks after the end of antibiotic treatment [104].

In accordance with ESCMID guidelines, CDI is defined as (i) > 3 unformed stools within 24 h or (ii) ileus or toxic megacolon in combination with evidence of toxin-producing *C. difficile* in stools and absence of another cause of symptoms, or (iii) pseudomembranous colitis diagnosed by endoscopy, colectomy, or histopathological examination [103]. In neutropenic patients, as well as in patients with severe colitis, diagnostic endoscopy is contraindicated because of the risk of colon perforation or hemorrhage [95].

The proper laboratory specimen is an unformed stool promptly submitted to the laboratory [5]. Processing a single specimen from a patient at onset of a symptomatic episode is sufficient and should not be repeated to avoid false positive results through multiple testing [5]. No single commercial test, but a two-step algorithm should be used to diagnose CDI from fecal samples. Possible combinations include a glutamate dehydrogenase (GDH) enzyme immunoassay (EIA) or a nucleic acid amplification test (NAAT) followed by a toxin A and B EIA [5].

To minimize the risk of developing CDI, antibiotics should cover a spectrum no broader than necessary and should be adapted with respect to results of cultures and/or susceptibility (**BIII**). If possible, antibiotics not intended for treatment of CDI should be discontinued after diagnosis of CDI [105–107] (**AII**); however, in febrile neutropenia, this may not always be possible.

There is insufficient evidence to recommend administration of prophylactic antibiotics or probiotics in cancer patients at risk for CDI [59, 94, 108] (**CIII, CII**_**t,**__**r**_ **)**. With the registration of bezlotoxumab, an antibody against *C. difficile* toxin B which is added to standard treatment and significantly decreases the likelihood of CDI recurrence, a new option for the secondary prevention of CDI in high-risk patients after an initial episode or first recurrence of CDI (**BII**_**t**_) or multiple recurrences (**AII**_**t**_ **)** has become available [109]. In patients still suffering from recurrent CDI after treatment with vancomycin and fidaxomicin, fecal microbiota transfer (FMT) as secondary prophylaxis may be discussed as an intervention of last resort [110–112] (**AII**_**t**_ **)**. While the current basis of evidence does not suggest safety issues specific to cancer patients [111], administration during neutropenia should be avoided, whenever possible (Table 4).

**Table 4:** Prevention and treatment of *Clostridium difficile* infection

Clinical situation	Intention	Intervention	SoR	QoE	Reference	Comments
Increased risk of CDI during antimicrobial treatment	Primary prevention	Antimicrobial prophylaxis	C	III	[94, 108]	
Increased risk of CDI during antimicrobial treatment	Primary prevention	Probiotic prophylaxis	C	II_r,t_	[59]	Insufficient data in immunocompromised patients
CDI—first episode or first recurrence	Secondary prevention	Bezlotoxumab 10 mg/kg qd iv	B	II_t_	[109]	
CDI—multiple recurrences	Secondary prevention	Bezlotoxumab 10 mg/kg qd iv	A	II_t_	[109]	
		Fecal microbiota transfer	A	II_t_	[110–112]	Only in case of recurrence after treatment with vancomycin and fidaxomicin
Diarrhea with CDI suspected—non-severe disease	Cure	Empirical therapy	C	II_u_	[113]	
Diarrhea with CDI suspected -Severe or complicated clinical disease	Cure	Empirical therapy	B	III		Only if patient instable and high suspicion of CDI
CDI—non-severe	Cure	Vancomycin 125 mg qid po for 10 days *or*	A	I	[114, 115]	
		Fidaxomicin 200 mg bid po for 10 days *or*				
		Metronidazole 400 mg tid po for 10 days	B	II_t_	[116–118]	If metronidazole 400 mg tablets not available, use 375 mg qid po
CDI—non-severe, oral administration not possible	Cure	Metronidazole 500 mg tid iv for 10 days	A	II_u_	[119, 120]	
CDI—severe	Cure	Vancomycin 125 mg qid po for 10 days *or*	A	II_t_	[114, 115, 121]	
		Fidaxomicin 200 mg bid po for 10 days				
		Metronidazole	D	I	[122]	
CDI—sever and oral administration not possible	Cure	Metronidazole 500 mg tid iv for 10 days	A	II_u_	[119, 120]	
		*plus* vancomycin 500 mg intracolonic every 4–12 h *and/or* vancomycin 500 mg qid by nasogastric tube	C	III	[123]	
CDI—refractory	Cure	Combination treatment with vancomycin po plus metronidazole any route *or*	C	II_h_	[124, 125]	
		Teicoplanin 100 mg bid po *or*		II_u_	[126]	
		Tigecyclin 100 mg loading, followed by 50 mg bid for 3-21d *or*		II_h_	[127–129]	
		Fecal microbiota transfer		II_u_	[110, 111]	
CDI—1st recurrence	Cure	Repeat strategy from 1st episode	C	III	[130, 131]	
		Vancomycin 125 mg qid po for 10 days *or*	A	II_t_	[114, 115]	
		Fidaxomicin 200 mg bid po for 10 days *or*				
		Vancomycin pulsed/taper strategy^a^			[132–134]	
CDI—multiple recurrences	Cure	Fidaxomicin 200 mg bid po for 10 days *or*	A	II_t_	[114, 115]	
		Vancomycin pulsed/taper strategy^a^			[132–134]	

*SoR* strength of recommendation, *QoE* quality of evidence; ^a^e.g., vancomycin 125 mg qid po for 7 to 14 days, 125 mg bid po for 7 days, 125 mg qd po for 7 days, 125 mg qd po every other day, 125 mg qd po every 3 days for 14 days

Results from one small, monocentric observational study in a mixed patient population do not suffice to generally recommend empirical therapy in patients with diarrhea and at risk for CDI [113] (**CII**_u_). However, in patients with symptoms compatible with CDI and severe or complicated disease, empirical treatment may be considered (**BIII**). Antiperistaltic agents, including opiates, are discouraged [135] (**DII**_u_).

For non-severe CDI, pooled data of all published randomized controlled trials suggests only a trend towards superior cure rates for oral vancomycin as opposed to metronidazole. However, recent findings indicate an increase in isolates resistant to metronidazole as opposed to vancomycin [116–118] (**BII**_**t**_). In two large randomized controlled trials, fidaxomicin met non-inferiority criteria when compared to vancomycin for treatment of non-severe and severe CDI [114, 115] (**AI**). Metronidazole should not be used in severe cases [122] (**DI**).

Metronidazole 500 mg tid iv for 10 days is likely to result in effective concentrations in feces and colon and may be an option if oral antimicrobials cannot be administered [119, 120] (**AII**_**u**_).

In severe cases of CDI, additional administration of vancomycin (e.g., 500 mg) by nasogastric tube and/or by rectal catheter may be discussed [123] (**CIII**). Refractory CDI is defined as lack of clinical response to standard CDI treatment, i.e., vancomycin and fidaxomicin, and should be distinguished from recurrent CDI. For this scenario, no data from randomized controlled trials is available. Therefore, alternatives including combination treatment [124, 125], treatment with teicoplanin [126], tigecycline [127–129], or an FMT [110, 111] cannot be recommended without reservations, due to lack of data (**CII**). In case of complicated CDI, a surgical evaluation should be obtained at an early stage of disease. However, surgical intervention in the neutropenic and/or thrombocytopenic patient should be reserved to selected complicated cases (**BIII**). In patients with a first recurrence, previous guidelines have recommended repetition of the initial strategy as the treatment of choice [103]. However, recent findings suggest a key role of the fecal microbiota in the pathophysiology of recurrent CDI [136]. Multiple treatments with standard vancomycin regimens seem to decrease the likelihood of long-term stabilization of these cases, as the diversity of the fecal microbiota is further compromised [130, 137]. In this setting, repetition of the initial strategy does no longer seem warranted (**CIII**). Alternatively, vancomycin 125 mg qid po for 10 days [114, 115] seems still warranted, if metronidazole was used during the initial episode. Fidaxomicin 200 mg bid po [114, 115] or a vancomycin pulsed/taper [132–134] strategy should be used, if standard vancomycin was used during the initial episode (**AII**_**t**_ **)**.

##### Other bacterial infections causing diarrhea (non-typhoidal *Salmonella*, *Shigella*, *Yersinia*, and *Campylobacter* spp.)

In cancer patients, infection-related diarrhea due to non-typhoidal *Salmonella*, *Shigella*, *Yersinia*, or *Campylobacter* spp. (SSYC) is a rare event (0–2.8%) [4, 138–140]. Clinical signs and symptoms include watery, mucoid, or bloody diarrhea; abdominal tenderness; fever; and nausea. Abdominal pain tends to be particularly severe in *Campylobacter* enteritis and may mimic appendicitis in *Yersinia* spp. and *Campylobacter* spp. infection. Since SSYC are typically community-acquired, testing for these pathogens should be restricted to fecal samples taken within 72 h of hospital admission from symptomatic patients. In case of clinical deterioration, an abdominal ultrasound or x-ray may be performed to detect an ileus or toxic megacolon. A thickened bowel wall may be detected by abdominal ultrasound or CT scan. In this case, the differential diagnosis of NEC should be considered. Perforation rarely occurs in this setting and may be identified by plain abdominal x-ray or abdominal CT scan.

Based on the low incidence of these infectious agents and the possibility of induction of resistance, prophylactic treatment is not recommended [4, 138–141] (**DII**_**t,u**_). While non-severe cases of diarrhea caused by bacteria other than *C. difficile* may not always require antibiotic treatment, severely ill and/or immunocompromised individuals should receive systemic treatment (**BIII**). Given the limited data in these populations, treatment recommendations for cancer patients were derived from studies performed in immunocompetent individuals. Immunocompromised patients suffering from non-typhoidal salmonellosis may benefit from therapy with ciprofloxacin. Alternatively, ceftriaxone iv may be administered depending on in vitro susceptibility test results [142] (**BIII**). In patients with *Salmonella* spp. bacteremia, treatment with a combination of ceftriaxone plus ciprofloxacin is recommended to avoid initial treatment failure before resistance test results are available and allow de-escalation to a monotherapy [142, 143] (**BIII**). Two randomized controlled trials on the treatment of shigellosis established ciprofloxacin or another fluoroquinolone as the treatment of choice with azithromycin being an effective alternative [144, 145] (**BII**_**t**_).

For infections with *Campylobacter* spp., azithromycin has become the drug of choice due to an increase in fluoroquinolone resistance [146] (**BII**_**t**_). Treatment with erythromycin is not considered standard of care, due to its unfavorable toxicity profile. Furthermore, a study performed in a pediatric population showed inferiority of erythromycin to azithromycin [147].

For infections caused by *Yersinia* spp*.*, treatment with a fluoroquinolone or trimethoprim-sulfamethoxazole is suggested (**BIII**). For patients with severe disease, the preferred regimen is a third-generation cephalosporin combined with gentamicin [148] (**BIII**). If feasible, antibiotic treatment in patients with Shigatoxin-producing *Escherichia coli* should be avoided. However, if an accompanying infection requires treatment, a carbapenem or azithromycin should be preferred, if suitable [149, 150] (**CIII**). Table 5 summarizes these recommendations.

**Table 5:** Treatment of non-typhoidal *Salmonella*, *Shigella*, *Yersinia*, and *Campylobacter* spp. (SSYC)

Clinical situation	Intention	Intervention	SoR	QoE	Reference	Comments
Neutropenia or immunosuppression	Prevention	Antimicrobial prophylaxis against *Salmonella*, *Shigella*, *Yersinia* or *Campylobacter* spp.	D	II_t,u_	[4, 138, 140]	
Diarrhea caused by non-typhoidal *Salmonella* spp.	Cure	Ciprofloxacin 500 mg bid po or 400 mg bid iv *or*	B	III		-Treat only if patient currently immunocompromised or severely ill -Consider local resistance patterns -Treatment duration recommended for immunocompetent patients is 5–7 days and should be extended to 14 days in immunocompromised individuals
		Ceftriaxone 2 g qd iv				
Bacteremia caused by non-typhoidal *Salmonella* spp.	Cure	Ceftriaxone 2 g qd iv *plus* ciprofloxacin 400 mg bid iv	B	III		Start with combination therapy and de-escalate once resistance data becomes available
Diarrhea caused by *Shigella* spp.	Cure	Fluoroquinolone, e.g., ciprofloxacin 400 mg bid iv *or* 500 mg bid po *or*	B	II_t_	[144, 145]	Treatment duration recommended for immunocompetent patients is 3–5 days and may be extended to 5–7 days in immunocompromised individuals
		Azithromycin 500 mg qd iv/po				
Diarrhea caused by *Campylobacter* spp.	Cure	Azithromycin 500 mg qd iv/po *or*	A B	II_t_	[145]	-Treat only if patient currently immunocompromised or severely ill -Treatment duration recommended for immunocompetent patients is 3 days and may be extended in immunocompromised individuals -High fluoroquinolone resistance
		Ciprofloxacin 400 mg bid iv or 500 mg bid po				
Diarrhea caused by *Yersinia* spp.	Cure	Ciprofloxacin 400 mg bid iv or 500 mg bid po *or* Trimethoprim–sulfamethoxazole 960/180 mg qd po/iv	B	III		-Treat only if patient currently immunocompromised or severely ill -Treatment duration recommended for immunocompetent patients is 5–7 days and may be extended in immunocompromised individuals
Bacteremia caused by *Yersinia* spp.	Cure	Ceftriaxone 2 g qd iv *plus* gentamicin 5 mg/kg qd iv	B	III	[148]	-Treat only if patient currently immunocompromised or severely ill -Treatment duration recommended for immunocompetent patients is 7–14 days and may be extended in immunocompromised individuals
Diarrhea caused by Shigatoxin producing *Escherichia coli*	Cure	Carbapenem iv or	C	III	[149, 150]	-Limited data in immunocompromised patients -If possible, restrict to supportive treatment, as antibiotics may be deleterious
		Azithromycin po				

*SoR* strength of recommendation, *QoE* quality of evidence

##### Viral infections

The most common viral causes of gastroenteritis in cancer patients include norovirus (earlier known as Norwalk-like virus), rotavirus, adenovirus, and cytomegalovirus (CMV). Self-limiting infections with norovirus and rotavirus may affect cancer patients of all risk groups. On the other hand, patients with impaired cellular immunity, e.g., due to a chronic lymphatic malignancy, immunosuppression after allogeneic stem cell transplantation (allo-SCT), treatment with alemtuzumab or other substances compromising T cell function, are at increased risk of developing clinically significant courses of viral gastroenteritis due to CMV or adenovirus, warranting treatment. These infections are unlikely to occur in patients undergoing conventional chemotherapy and those suffering from solid tumors [151–154].

Norovirus is a frequent cause of acute gastroenteritis during the cold season. Transmission occurs by contact with excretions, even in the form of aerosols, and requires only 10–100 viral particles. The incubation period of 12–48 h is typically followed by vomiting, diarrhea, abdominal pain, myalgia, and low fever. Incidence rates of 2.5 and 1.3%, respectively, have been reported from cohorts of neutropenic high-risk and allo-SCT patients presenting with diarrhea, respectively [4, 27, 155]. In the immunocompetent host, the course is self-limiting with symptoms commonly lasting 12–72 h and a mean duration of viral shedding of 4 weeks after onset of illness. In the immunocompromised patient, duration and intensity of clinical signs and symptoms and of viral shedding may be considerably prolonged [156, 157].

Real-time PCR (sensitivity 94%, specificity 92%) is currently considered the gold standard for the detection and typing of norovirus; numerous conventional and real-time norovirus RT-PCR assays have been developed [158, 159]. A number of EIAs are commercially available for the detection of norovirus antigens in stool specimens. EIA is a method for outbreak investigations, particularly in laboratories that lack molecular diagnostic capabilities [152].

A considerably high mortality rate of up to 25% has been attributed to norovirus gastroenteritis in allo-SCT patients [160]. No specific treatment options are currently available; therapeutic management is supportive.

Rotavirus infections are a far less common cause of gastroenteritis in the adult immunocompromised patient. A 3-day course of nitazoxanide significantly reduces the duration of rotavirus disease in immunocompetent pediatric patients [161, 162]. As this therapy has not been assessed in immunocompromised patients and only in very few adult patients [163], further studies are required before a recommendation can be made (**CII**_**t**_). In two patients, oral immunoglobulin has been successfully used to treat rotavirus gastroenteritis [164] (**CIII**).

Adenovirus is typically associated with gastroenteritis in newborns and children as well as with keratoconjunctivitis epidemica and acute respiratory distress syndrome. In patients with impaired cellular immunity, life-threatening courses of adenovirus disease have been reported [165, 166].

Cidofovir is approved for severe adenovirus infections, even though only limited data from case reports and small series is available. Low-dose cidofovir (1 mg/kg thrice a week) was effective in one adult patient [165], and in a report from a pediatric hematology unit with an adenovirus outbreak, seven patients were successfully treated with cidofovir 5 mg/kg iv once weekly for 2 weeks, then once every other week [167].

Treatment with cidofovir may therefore be discussed in severely ill patients with adenovirus-associated diarrhea (**BII**_**u**_); however, its considerable nephrotoxicity should be taken into account.

CMV is found in blood and excretions of individuals with profound and long-lasting cellular immunosuppression and is a rare cause of gastrointestinal infections in other patient groups [168–171].

Patients may present with nausea, vomiting, bloody or non-bloody diarrhea, fever, abdominal pain, and prolonged anorexia [172]. CMV infection (viremia) is diagnosed by detection of antigen (pp65; antigenemia assay), DNA, or mRNA. Quantification of viral load by PCR is also widely available [173]. However, for diagnosis of CMV disease with organ involvement, such as enteritis, detection of CMV in peripheral blood is not appropriate and may be negative. Similarly, CMV detection in fecal samples does not suffice to establish a diagnosis [174]. In addition, CMV detection in an endoscopically obtained biopsy specimen from suspicious areas in the esophagus, stomach, small bowel, or large intestine is needed [175, 176]. The diagnosis is made by the association of CMV disease with specific mucosa pathology and appropriate symptoms [177].

Recommendations on CMV prophylaxis and pre-emptive treatment are given in the updated European Conference on Infections in Leukemia (ECIL) recommendations on the management of CMV, HHV-6, HHV-7, and Kaposi-sarcoma herpesvirus (HHV-8) infections in patients with hematological malignancies or those after SCT [178, 179].

We recommend treating gastrointestinal CMV disease with ganciclovir for 2–3 weeks with induction dosing of 5 mg/kg bid iv, followed by several weeks of maintenance therapy at a dose of 5 mg/kg qd iv on 5–6 days per week. The prolonged treatment interval is intended to cover the period of mucosal re-epithelialization [172] (**AI**). The addition of intravenous immunoglobulins to antiviral therapy may be considered [180–182] (**CII**_**u**_). Concerning antiviral treatment alternatives, the administration of foscarnet [183] (**BII**_**t**_), cidofovir [184–186] (**BII**_**u**_), or the combination of foscarnet and ganciclovir may be considered [187–189] (**BII**_**t**_). Both substances, foscarnet and cidofovir, are associated with significant renal toxicity. Recommendations on the treatment of viral gastroenteritis are summarized in Table 6.

**Table 6:** Treatment of viral gastroenteritis

Clinical situation	Intention	Intervention	SoR	QoE	Reference	Comments
Rotavirus enteritis	Cure	Nitazoxanide 7.5 mg/kg bid po	C	II_t_	[161, 162]	Mainly assessed in immunocompetent pediatric patients
		Oral immunoglobulin	C	III	[164]	No sufficient evidence to recommend dosage
Adenovirus enteritis	Cure	Cidofovir 5 mg/kg iv once weekly for 2 weeks, then once every other week	B	II_u_	[165, 167]	To reduce cidofovir toxicity, add at least 2 L of iv prehydration and probenecid 2 g po 3 h prior and 1 g 2 and 8 h following cidofovir
CMV enteritis	Cure	Ganciclovir 5 mg/kg bid iv for 2–3 weeks followed by several weeks of 5 mg/kg qd iv on 5 days per week	A	I	[172]	
		Foscarnet 90 mg/kg bid iv over 2 h *or* 60 mg/kg tid iv over 1 h *or*	B	II_t_	[183]	Used in a pre-emptive setting
		Cidofovir 5 mg/kg iv once weekly for 2 weeks, then once every other week *or*	B	II_u_	[184–186]	To reduce cidofovir toxicity, add at least 2 L of iv prehydration and probenecid 2 g po 3 h prior and 1 g 2 and 8 h following cidofovir
		Foscarnet 90 mg/kg bid iv over 2 h or 60 mg/kg tid iv over 1 h *plus* ganciclovir 5 mg/kg bid iv for 2–3 weeks followed by several weeks of 5 mg/kg qd iv on 5 days per week	B	II_t_	[187–189]	Alternatively, the dosage of both combination partners may be reduced by 50%
		Addition of iv immunoglobulin	C	II_u_	[180–182]	No sufficient evidence to recommend dosage

*SoR* strength of recommendation, *QoE* quality of evidence

##### Parasitic infections

Given extensive travels, and growing populations of migrants, rising incidence rates of gastrointestinal infections with parasites are to be expected. In cancer patients with persistent diarrhea in spite of a complete work-up for bacterial and viral pathogens, examination of stools for parasites may therefore be warranted. Potentially causative pathogens include *Blastocystis* spp., Cryptosporidium, *Cyclospora cayetanensis*, *Entamoeba histolytica*, *Giardia lamblia*, *Isospora belli*, *Sarcocystis hominis*, *S. suihominis*, and *Strongyloides stercoralis.* For a diagnostic work-up, three fresh stool samples should be analyzed at a sufficiently qualified laboratory. Treatment options have not been specifically assessed in immunocompromised cancer patients and were extrapolated from studies in other patient populations. While many randomized controlled trials were performed in this area of research, most of them are of limited size or quality.

Based on results from a randomized controlled trial, metronidazole has been established as the treatment of choice for *Blastocystis* spp. infection [190] (**AII**_**t**_). Alternatives, the majority of them supported by only limited clinical evidence, include nitazoxanide, trimethoprim–sulfamethoxazole, tinidazole, and paromomycin [191–193] (**BII**_**t**_).

Nitazoxanide is the only FDA-registered drug for the treatment of *Cryptosporidium* spp. infections. Studies leading to registration were performed mainly in HIV-infected patients and children [194–197]. A meta-analysis performed for immunocompromised HIV and non-HIV patients confirmed improved oocyte clearance, but did not confirm any impact of nitazoxanide treatment on resolution of diarrhea. However, the subpopulation of HIV seronegative patients experienced a significant clinical benefit [198]. Similarly, data from a compassionate use program in which nitazoxanide was administered over variable periods of time to patients with HIV-related cryptosporidiosis does suggest a considerable clinical benefit [199] (**BII**_**r,t**_). As a non-registered alternative, paromomycin was assessed in the same meta-analysis, but no advantages over placebo treatment could be confirmed. However, all patients included into the analysis were suffering from advanced HIV, such that the efficacy in patients immunocompromised for other reasons is not evaluable [198]. In immunocompetent individuals, however, improvement in 60–70% of patients has been documented [197, 200] (**CII**_**r,t**_).

Intestinal *E. histolytica* amebiasis without dysentery (blood, mucus in stools) may be treated with paromomycin, an intraluminal agent, alone [201] (**BII**_**t**_). Patients with *E. histolytica* colitis should be treated with tinidazole (**AII**_**r,t**_) or metronidazole (**BII**_**r,t**_), followed by an intraluminal agent, e.g., paromomycin, diiodohydroxyquin, or diloxanide furoate (**BIII**), intended to eliminate intraluminal cysts [202].

Several randomized controlled trials support the use of 5-nitroimidazoles (metronidazole or tinidazole) [203, 204] or benzimidazoles (albendazole or mebendazole) [204] for the treatment of *Giardia* spp. infections (**AII**_**r,t**_). In case of resistance, nitazoxanide may be a suitable alternative [205] (**BII**_**t**_).

A small randomized trial in HIV-infected patients showed efficient treatment of *Isospora belli* or *Cyclospora* spp. infections by use of trimethoprim-sufamethoxazole (**AII**_**t**_) and alternatively oral ciprofloxacin [206, 207] (**BII**_**t**_).

A small number of patients with *Isospora belli* infection have also been treated successfully with nitazoxanide, but there is not sufficient evidence to recommend use [208] (**CIII**).

According to a recent Cochrane analysis, ivermectin (**AII**_**r,t**_) is superior to albendazole (**BII**_**r,t**_) in the treatment of infection with *Strongyloides stercoralis* [209].

Gastrointestinal disease with *Sarcocystis hominis* and *S. suihominis* is usually self-limiting and does therefore not require any treatment apart from supportive care*.* Table 7 summarizes all recommendations on the treatment of parasitic infections.

**Table 7:** Treatment of parasitic diarrhea/colitis

Clinical situation	Intention	Intervention	SoR	QoE	References	Comment
*Blastocystis* spp. Infection	Cure	Metronidazole 500 mg tid po for 7 days	A	II_t_	[190]	Treatment of colonization without compatible signs and symptoms not recommended
		Alternatives: nitazoxanide, trimethoprim–sulfamethoxazole, tinidazole, paromomycin	B	II_t_	[191–193]	
*Cryptosporidium* spp. Infection	Cure	Nitazoxanide 500 mg bid po for 3 days *or*	B	II_r,t_	[194–199]	Higher dosages (e.g., 1000 mg bid for up to 14 days) might be required in severely immunocompromised patients
		Paromomycin 25 to 35 mg/kg/day po in 2 to 4 divided doses for 10 to 14 days	C	II_r,t_	[197, 198, 200]	
*Cyclospora* spp. Infection	Cure	Trimethoprim–sulfamethoxazole 160/800 mg bid po for 7 days *or*	A	II_t_	[206, 210, 211]	
		Ciprofloxacin 500 mg bid po for 7 days	B	II_t_		
*Entamoeba histolytica* infection, non-invasive	Cure	Paromomycin 30 mg/kg qd po in three divided doses for 7 days	B	II_t_	[201]	
*Entamoeba histolytica*, invasive colitis	Cure	Tinidazole 2 g qd po for 3 days *or*	A	II_r,t_	[202]	
		Metronidazole 500 mg tid po for 10 days followed by:	B	II_r,t_	[202]	
		Paromomycin 25–30 mg/kg qd po in three divided doses for 7 days *or*	B	III	[202]	
		Diiodohydroxyquin 650 mg tid po for 20 days *or*				
		Diloxanide furoate 500 mg tid po for 10 days				
*Giardia* spp. Infection	Cure	Metronidazole 250 mg tid po for 5–10 days *or*	A	II_r,t_	[203, 204]	
		Tinidazole 2 g po as a single dose				
		Albendazole 400 mg qd po for 5–10 days *or*	A	II_r,t_	[204]	
		Mebendazole 200 mg tid po for 3–7 days				
		Nitazoxanide 500 mg bid for 3 days	B	II_t_	[205]	Higher dosages (e.g., 1000 mg bid for up to 14 days) might be required in severely immunocompromised patients
*Isospora belli* infection	Cure	Trimethoprim–sulfamethoxazole 160/800 mg bid po for 7 days *or*	A	II_t_	[206, 207]	
		Ciprofloxacin 500 mg bid po for 7 days *or*	B	II_t_	[206]	
		Nitazoxanide 500 mg bid po for 7 days	C	III	[208]	
*Strongyloides stercoralis* infection	Cure	Ivermectin 200 μg/kg qd po for 2 days	A	II_r,t_	[209]	
		Albendazole 400 mg bid po for 3–10 days	B	II_r,t_		
